# Immune Reconstitution Inflammatory Syndrome and the Influence of T Regulatory Cells: A Cohort Study in the Gambia

**DOI:** 10.1371/journal.pone.0039213

**Published:** 2012-06-20

**Authors:** Irfan Zaidi, Kevin Peterson, David Jeffries, Hilton Whittle, Thushan de Silva, Sarah Rowland-Jones, Assan Jaye, Bouke C. de Jong

**Affiliations:** 1 Medical Research Council (UK) Unit, Fajara, The Gambia; 2 Institute for Tropical Medicine, Antwerp, Belgium; 3 Weatherall Institute of Molecular Medicine, Medical Research Council Human Immunology Unit, John Radcliffe Hospital, Oxford, United Kingdom; 4 Division of Infectious Diseases, New York University, New York, New York, United States of America; Rush University, United States of America

## Abstract

**Objective:**

The factors associated with the development of immune reconstitution inflammatory syndrome in HIV patients commencing antiretroviral therapy have not been fully elucidated. Using a longitudinal study design, this study addressed whether alteration in the levels of T regulatory cells contributed to the development of IRIS in a West African cohort of HIV-1 and HIV-2 patients. Seventy-one HIV infected patients were prospectively recruited to the study and followed up for six months. The patients were categorized as IRIS or non-IRIS cases following published clinical guidelines. The levels of T regulatory cells were measured using flow cytometry at baseline and all follow-up visits. Baseline cytokine levels of IL-2, IL-6, IFN-γ, TNF-α, MIP-1β, IL-1, IL-12, IL-13, and IL-10 were measured in all patients.

**Results:**

Twenty eight percent of patients (20/71) developed IRIS and were predominantly infected with HIV-1. Patients developing IRIS had lower nadir CD4 T cells at baseline (p = 0.03) and greater CD4 T cell reconstitution (p = 0.01) at six months post-ART. However, the development of IRIS was not influenced by the levels of T regulatory cells. Similarly, baseline cytokine levels did not predict the onset of IRIS.

**Conclusion:**

The development of IRIS was not associated with differences in levels of T regulatory cells or baseline pro-inflammatory cytokines.

## Introduction

Immune reconstitution inflammatory syndrome (IRIS) results from an exuberant immune response against residual antigens (paradoxical IRIS) or viable pathogens (unmasking IRIS) in HIV infected patients commencing antiretroviral therapy [1]–[3]. While most IRIS develops in response to mycobacteria, a number of other pathogens have also been associated with the development of IRIS [4]. IRIS due to all causes develops in 3% to 39% of patients who start ART, and TB-IRIS in 3% to 43% [5]. In two prospective South African cohorts, all-cause IRIS developed in 10% of patients starting ART, and TB-IRIS developed in 23% of patients who had started TB therapy prior to ART [6], [7]. The peak incidence of IRIS occurs 2 to 8 weeks after ART initiation, and both paradoxical and unmasking IRIS are more common in patients with a lower nadir CD4 prior to starting ART [8], [9]. While IRIS often has a benign course, it can cause considerable morbidity and occasionally be fatal [10].

The etiology of IRIS remains enigmatic, including its immunological mechanism and predictors. Increased proliferation and production of IFN-γ from highly activated CD4 T cells towards tuberculin, and increased levels of KIR- γδ^+^ T cells were observed in TB- IRIS compared to non-IRIS ART controls [11], [12]. A more recent study demonstrated that IRIS patients displayed higher levels of HLA-DR^+^, Ki-67^+^, PD-1^+^ and effector memory subsets of CD4 T cells compared to non-IRIS controls [13]. Similarly, using a model of Mycobacterium avium (M. avium) challenge in lymphopenic mice, Barber et al., demonstrated the onset of symptoms consistent with IRIS after injection of exogenous CD4 T cells [14]. Similarly, the levels of activated CD8 T cells are elevated in IRIS patients compared to ART controls, suggesting that these cells may also contribute to the development of IRIS [2], [15].

T regulatory cells (Tregs) are committed suppressors of the immune system. While multiple subtypes have been described, the best characterized remain the natural Tregs, which originate in the thymus yet can also be induced in the periphery. Besides CD4 and CD3, these cells are defined by the constitutive expression of the transcription factor Forkhead Box protein 3 (FOXP3) [16]–[17]. Tregs function by suppressing the secretion of cytokines and proliferative responses of a variety of immune cell types [18]–[19] in response to microbial and self-antigens [20]–[21]. Previous studies analyzing the role of Tregs in IRIS conducted using a cross-sectional study design did not show any differences in their levels between IRIS and non-IRIS cases [22]–[23]. However, Seddiki et al., reported that the suppressive ability of Tregs in M.avium IRIS cases was lower than that observed in non-IRIS cases [23].

The clinical resemblance of IRIS to paradoxical reactions, or a hyperactive immune system, suggests that the balance between immune activation and regulation is disturbed early on during ART. Using a prospective longitudinal study design we focused on the dynamics of Tregs following ART initiation and tested the hypothesis that (all-cause) IRIS results from a relative delay in the reconstitution of FOXP3 positive T regulatory cells. In addition, we assessed whether baseline plasma cytokines could predict the occurrence of IRIS after initiation of ART. The results from this study argue against a significant role for Treg levels, either proportions or absolute numbers, as predictors of the development of IRIS.

## Results

### Incidence of IRIS in the Study Cohort

Of 80 patients enrolled after informed consent, 71 (89%) completed more than 12 weeks of ART without an interruption of greater than one week. One patient was found to have CD4 count >800 cells/µl twice on repeat baseline testing, and was subsequently taken off ART. Four patients were lost to follow-up before 12 weeks, and 2 patients interrupted ART for 1 month. Three patients died, of whom two had interrupted ART for more than four weeks prior to death without IRIS symptoms recorded at the last visit. One patient, in whom IRIS could not be excluded, died after 8 weeks on ART.

**Figure 1:**
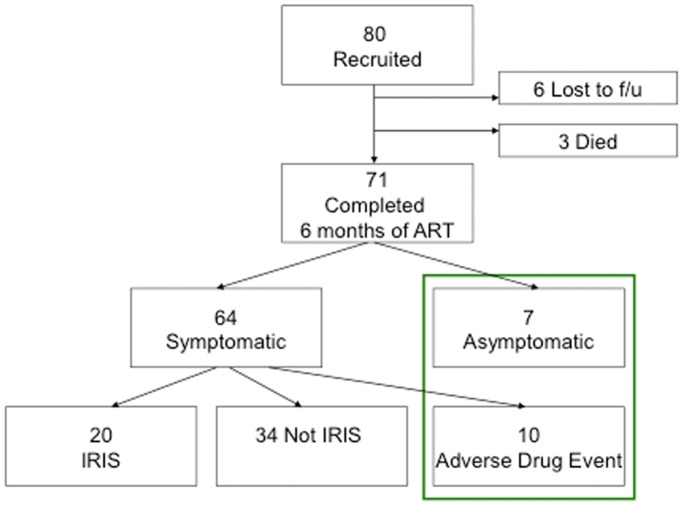
Classification of patients recruited to the study according to IRIS status.

Of the 71 patients included in the analysis, 21 (30%) developed symptoms consistent with IRIS using the criteria set out by Haddow et al [7] (Figure1).

**Figure 2:**
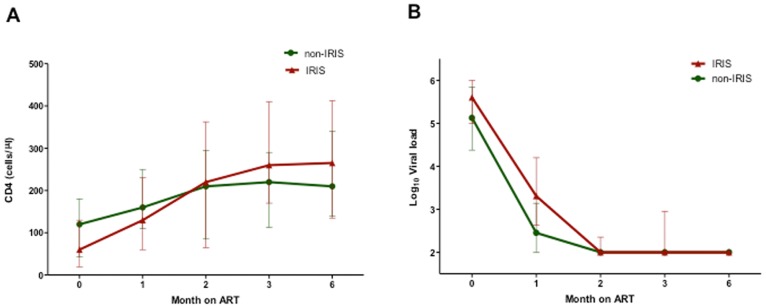
Comparison of CD4 and Viral load levels in IRIS and non-IRIS patients. A) CD4 levels (cells/µl) and B) Viral loads (copies/ml) after initiation of ART in study subjects classified by IRIS status. The error bars represent the interquartile ranges.

IRIS patients typically had highly advanced disease and commenced therapy with a significantly lower nadir CD4 count than non-IRIS patients (60 versus 120 cells/µl, p = 0.03, Figure 2). However, the IRIS group also showed a significantly greater increase in absolute CD4 T cells levels compared with non-IRIS group six months after commencement of antiretroviral therapy (150 versus 100 cells/µl, p = 0.04). All subjects showed excellent responses to treatment and 92% of patients had undetectable viral loads at the end of the follow-up period (Table 1). The majority of patients that developed IRIS showed symptoms within the first month after initiation of ART (16 of 20 (80%)). The median duration of IRIS symptoms was 49 (28–91) days. Pathogens were rarely identified, while the lung and skin/mucosal surfaces were involved in most cases (Table 2). The combination of fever and cough with or without other symptoms was present in half of the cases, and 5 of these 8 patients were among the 6 that had a history of completing TB treatment prior to starting ART.

**Table 1:** Patient characteristics.

	All	IRIS	Non-IRIS	P value
**N (%)**	71	20 (28%)	51 (72%)	
**Male sex**	25 (35%)	5 (24%)	20 (39%)	
**Age**(median)	41	39	43	ns
**CD4 nadir** (median)	100(30–160)	60(20–130)	120(45–180)	0.03
**CD4 increase in 6 mo**(median)	120(70–205)	150(120–330)	100(50–180)	0.01
HIV type				
1	58 (82%)	17 (85%)	41 (80%)	ns
2	9 (13%)	2 (10%)	7 (14%)	ns
dual	4 (6%)	1 (5%)	3 (6%)	ns
**VL <100 copies/ml**	65 (92%)	18 (90%)	47 (92%)	
**Ethnicity**				
Mandinka	28 (39%)	7 (30%)	21 (41%)	ns
Wolof	9 (13%)	2 (10%)	7 (14%)	ns
Fula	8 (11%)	3 (10%)	5 (10%)	ns
Jola	8 (11%)	3 (10%)	5 (10%)	ns
Other	18 (25%)	5 (20%)	13 (25%)	ns
**ART 24 wk**	61 (86%)	19 (95%)	42 (69%)	ns

IRIS = Immune reconstitution inflammatory syndrome.

ADE = Adverse drug effects.

VL = Viral load, <100 = undetectable within first 9 months of ART.

ART = Antiretroviral therapy.

ART 24 wk = No interruption in ART use >3 days over 24 weeks.

ns = not significantly different, p>0.05.

**Table 2:** Clinical features of IRIS cases.

HIV Type	Prior TB	Time toIRIS (wks)	IRIS duration(days)	Predominant organ	Clinical finding	Diagnostic results	Admitted	nadir VL	CD4 change (%)
1	NO	2	13	SKIN	Lip Ulceration	None	NO	<100	80(12%)
1	YES	4	14	PULMONARY	Fever, Cough, Headache	None	NO	<100	120(7%)
1	YES	2	14	PULMONARY	Fever, Cough, cervical LAD, anal warts	AFB neg LN	YES	<100	410(9%)
1	YES	2	28	PULMONARY	Fever, Cough, Headache	RUZ & RMZ infiltrate, sputum AFB-, TB Tx response+	YES	<100	350(6%)
1	NO	3	30	SKIN	Lip Ulceration	None	NO	5014	580(27%)
1+2	NO	1	32	SKIN	Herpes zoster at wk 10	Mantoux 40 mm	NO	<100	150(9%)
1	NO	7	35	LYMPH	Submandibular LAD	RLZ infiltrate and LAD	NO	<100	90(5%)
1	NO	4	40	PULMONARY	Fever, cough	Patchy RLZ infiltrate	NO	<100	240(10%)
1	NO	2	42	PULMONARY	Fever, sweats, cough	None	NO	<100	330(15%)
1	NO	4	49	SKIN	Tongue and perianal ulceration	None	NO	<100	210(10%)
2	YES	4	49	SKIN	Fever,suppurative ulcerations on legs with cellulitis	Gram: GPC in clusters, culture S. aureus	NO	<100	330(9%)
1	YES	2	66	RENAL/PULMONARY	Fever,cough, flank and inguinal pain	UA with nephritic sediment	YES	<100	390(12%)
1	YES	3	67	PULMONARY	Fever, cough	Diffuse R infiltrate(unilateral pleural effusion)	YES	<100	60(15%)
1	NO	2	70	SKIN	vulvar ulceration with LAD	None	NO	<100	300(11%)
1	NO	12	84	LYMPH	cervical LAD	None	NO	<100	90(3%)
1	NO	8	88	PULMONARY	Fever, Cough, Headache	RLZ infiltrate	NO	<100	280(13%)
1	YES	3	91	SKIN	Fever,HA, ulcer, generalized rash	None	NO	<100	50(5%)
1	YES	1	91	PULMONARY	Fever, cough,extensive folliculitis	Diffuse L infiltrate,AFB pos sputum	YES	<100	130(11%)
2	NO	3	110	PULMONARY	Fever, cough	None	NO	<100	150(8%)
1	NO	12	296	SKIN	Fever, cough,extensive molluscum	None	NO	985	110(2%)

UA = urinalysis, RLZ = right lower zone, GPC = Gram positive cocci, RUZ = right upper zone, RMZ = right middle zone, LN = lymph node, LAD = lymphadenopathy, HA = Headache.

### T Regulatory Cell Reconstitution in Patients with and without IRIS

**Figure 3:**
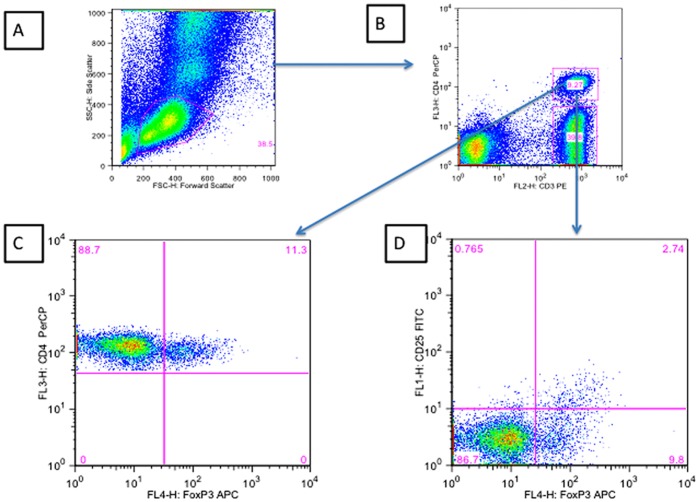
Gating strategy employed to determine the percentage of CD3^+^CD4^+^FOXP3^+^ and CD3^+^CD4^+^CD25^+^FOXP3^+^ cells. A) Forward Scatter vs Side Scatter B) CD3 versus CD4 within the lymphocyte gate C) FOXP3 versus CD4 within the CD4^+^CD3^+^ gate D) FOXP3^+^ versus CD25^+^ within the CD3^+^CD4^+^ gate.

**Figure 4:**
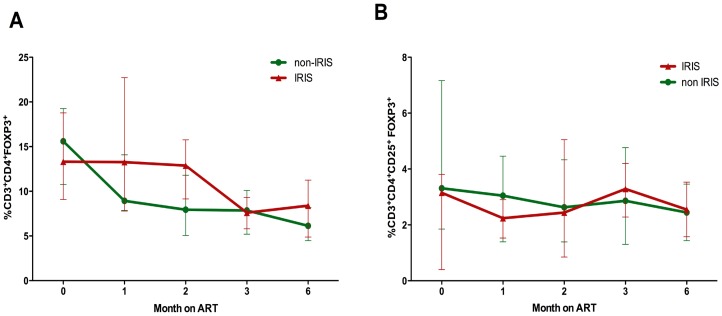
Change in T regulatory cells in IRIS and non-IRIS patients after the initiation of antiretroviral therapy. A) Percentage of total CD3^+^CD4^+^FOXP3^+^ T cells or B) CD3^+^CD4^+^CD25^+^FOXP3^+^ after initiation of ART till six months post ART. The trend lines denote the median percent values for each group over the 6-month study period.

Tregs were defined as CD4 T cells co-expressing the transcription factor FOXP3. The gating strategy employed to enumerate Tregs as a percentage of total CD4 T cells is shown in Figure 3. An alternative definition that included CD25^+^ as part of the definition of Tregs was also used for comparison (Figure 4).

The percent Treg levels expressed as CD4^+^CD4^+^FOXP3^+^ were similar between the IRIS (13.31%, 95% CI: 9.1–19.2) and non-IRIS group (15.6%, 95% CI: 11.3–18.9) at baseline, while the absolute levels of Tregs were lower in the IRIS group (6.3 cells/l, 95%CI: 2.4–16.2) versus the non-IRIS group (14.8 cells/ul, 95%CI: 5.2–28.5), although this lost significance after adjusting for total CD4 levels (p  = 0.08, data not shown).

The median percentage of Tregs declined after initiation of ART in the non-IRIS group but remained high in the IRIS group until three months post ART initiation. At six months post ART initiation, the percentage of Tregs had declined to significantly lower levels than at baseline in both the IRIS and non-IRIS groups equally (Figure 4A). Generalized estimating equations were used to analyze whether the magnitude or trend of Tregs differed between the IRIS and non-IRIS groups during the six-month study period. The trend of Tregs was not significantly different between the two groups and this was irrespective of whether Tregs were defined as CD3^+^CD4^+^FOXP3^+^ (p = 0.1452) or CD3^+^CD4^+^CD25^+^FOXP3^+^ (p  = 0.2815) (Figure 4A and 4B).

### Baseline Cytokines and Chemokines do not Predict IRIS

Plasma cytokine levels were measured at baseline to determine whether these could be used to predict the onset of IRIS. The levels of IL-12, IL-13, and IL-17 were below the limit of the assay in all patients tested. Plasma levels of IL-2, IL-6, IFN-γ MIP-1β, IL-10 and TNF-α were comparable in all groups (Table 3). There was no significant correlation observed between the levels of CD4^+^FOXP3^+^ cells and any of the cytokines measured.

**Table 3:** Comparison of cytokine levels between IRIS and non-IRIS patients before commencing ART. The interquartile ranges are displayed in brackets beside median values for each group and cytokine measured.

	Analyte (pg/ml)					
IRIS category	IL-2	IL-6	IFN-γ	MIP-1β	TNF-α	IL-10
Non-IRIS	0.765	7.4	10.65	73.65	0	2.84
	(0–12.27)	(3.16–10.83)	(4.57–34.66)	(54.11–124.91)	(0–1.67)	(1.21–4.22)
IRIS	4.86	11.16	27.11	72.52	2.1	4.38
	(0–28.74)	(3.84–25.47)	(4.57–243.78)	(56.49–98.21)	(0–4.17)	(1.45–9.98)
p value	0.275	0.45	0.09	0.34	0.845	0.65

## Discussion

IRIS remains elusive, both as a clinical phenotype with imprecise case definition, and as an immunological phenomenon during the massive overhaul of the immune system as people start ART. Several case definitions have been proposed for IRIS, both TB-IRIS [24] and all-cause IRIS [7]. We applied the most recent all-cause IRIS case definition used in many other studies [7].

The incidence of IRIS (28%) in our study was comparable with a similar study in South Africa, Mozambique and lower than reported in Senegal [5], [25]. IRIS patients had significantly lower nadir CD4 T cell counts and showed a greater recovery of CD4 T cell counts compared with the non-IRIS group. These results re-affirm that a low CD4 T cell count at the time of initiating ART is a risk factor for the development of IRIS [4], [9], [26], [27].

The majority of IRIS cases developed within the first month after starting on ART. In addition the IRIS patients showed a higher recovery of CD4 T cells compared to the non-IRIS group. These findings reiterate the need for commencing ART early and for close monitoring of HIV infected patients who initiate ART. Unique to this study, approximately 10% of subjects included were infected with HIV-2. These individuals showed similar dynamics in viral suppression and CD4 reconstitution to HIV-1 patients, and two of these patients developed IRIS. This emphasizes the point that in HIV-2 infected individuals who progress to AIDS, the clinical course often mirrors that of HIV-1 infected individuals.

Deciphering the immunopathogenesis of IRIS remains a challenge. Higher levels of immune activation prior to commencement of ART have been identified as a risk factor for development of IRIS [11]–[12]. Hence, we hypothesized that Treg dynamics in patients commencing ART could distinguish IRIS versus non-IRIS cases. Although we noted lower levels of absolute Tregs at baseline in those patients who later developed IRIS, percentage levels of Tregs were similar in both groups of patients. The proportion of Tregs in all patients declined steeply after the initiation of ART in all patients, which is consistent with the results of a recently published study [28]. However there appeared to be a slower decline in Treg levels in the IRIS group, and persistently higher Tregs after the occurrence of IRIS could be a compensatory mechanism for the inflammatory syndrome. Due to the high variability these were not significantly different from the non-IRIS group. However, we cannot exclude the possibility that Treg dynamics may differ within the first month (wherein the majority of IRIS cases develop) after initiation of ART between IRIS and non-IRIS cases.

Our results are consistent with a previous study comparing Treg levels in TB patients commencing ART, which similarly showed no significant differences in the percentage of Tregs in TB-IRIS at the time of IRIS presentation versus non-IRIS cases two weeks post ART treatment [22]. Neither the TB-IRIS study nor our own included functional assays. A cross-sectional study on 8 patients with Mycobacterium avium-related IRIS identified high numbers of Tregs which were unable to suppress the secretion of pro-inflammatory cytokines IFN-γ, TNF-α and IL-6 in vitro [23]. It is therefore feasible that despite high frequencies of Tregs, IRIS may result from inadequate suppressive function of these cells. Further studies that quantitate Treg function at ART initiation and prior to development of IRIS can clarify whether Treg dysfunction rather than frequencies indeed contributes to IRIS.

It appears that the mechanism of IRIS in patients commencing anti-retroviral therapy, including the role of T-cells, differs by pathogen [29]–[30]. Treg levels specifically were not significantly different between TB-IRIS cases and non-IRIS cases in two studies [22]–[23], while Tregs were elevated in cryptococcal meningitis-IRIS cases [31]. Since we were unable to determine the pathogen responsible for development of IRIS in most of our patients, we can only speculate that the lack of association between Treg levels and IRIS may be a result of heterogeneity in the causative pathogens in the IRIS patients.

Higher levels of pro-inflammatory cytokines before resumption of ART have been linked to a greater risk of developing IRIS [32]–[33]. Even though there were higher median levels of IL-6 and IFN-γ in the IRIS group compared to the non-IRIS group at baseline, these differences were small and did not predict the development of IRIS.

In summary the results of this study show that neither Treg levels nor cytokine levels predict the onset of all-cause IRIS. Further investigation is required to assess Treg function, in order to determine whether qualitative rather than quantitative Treg differences play a role in the development of IRIS.

## Methods

### Clinical

In a prospective observational cohort conducted at the MRC Laboratories in The Gambia, consecutive ART naïve adult African patients with a CD4 count below 200, scheduled to commence ART, were recruited after giving informed consent. We included people infected with HIV-1, HIV-2, or dual infection. Patients were screened for TB at baseline with symptom review and chest radiographs. They were encouraged to return to the clinic anytime they developed new symptoms. On their scheduled return visits at 2, 4, 8, 12, and 24 weeks post ART initiation, patients were asked IRIS specific questions prior to seeing a physician, including whether they had a fever, night sweats, cough, headache, nausea, abdominal pain, weakness, visual problems, skin lesions, or any swelling. The treating physicians assessed each patients’ likelihood of having IRIS, and all cases were subsequently reviewed and classified as IRIS based on the criteria outlined by Haddow et al [7].

### Laboratory

Patients had viral load, full blood count and lymphocyte subsets determined as part of their routine medical care at baseline, 12 and 24 weeks. In addition, patients donated blood for flow cytometry analysis at baseline and the routine follow-up visits at 4, 8, 12 and 24 week visits. Plasma was stored at −20°C.

### Enumeration of Tregs using Whole Blood FACS Staining

Fresh whole blood (150 µl) was stained within 6 hours using conjugated monoclonal antibodies to CD25-FITC, CD4-PerCP, CD3-PE (Becton Dickinson, USA) and FOXP3-APC (Clone: PCH101, Ebiosciences, USA) using the recommended intracellular staining protocol for FOXP3. Single stains for CD8 were used to adjust compensations before acquisition of samples on a 4-colour FACS Calibur. A minimum of 150,000 events were collected in the PBMC gate. The data were analyzed for percent of CD3+CD4+ as well as CD3+CD4+CD25+ T cells expressing FOXP3, using the FlowJo software.

#### CD4 T cell counts

Enumeration of CD4 levels (CD4% of total CD3 T cells) was done using BD MultiTest reagents and MultiSet software (BD Biosciences, USA) as recommended by the manufacturers.

#### Viral load measurements

HIV-1 and HIV-2 plasma viral loads were quantified using a PCR based method [34]–[35] using primers targeted to the viral LTRs and quantitative detection of PCR products by enzyme-linked oligonucleotide binding. The assay has a lower limit of detection of 100 RNA copies/ml.

#### Measurement of plasma cytokine levels

Plasma levels of IL-2, IL-6, IL-10, IL-12, IL-13, IL-17, TNF-α, MIP-1β and IFN-γ were measured at baseline using a single Bioplex assay (Bio-Rad, USA) on undiluted plasma using standards and controls as recommended by the manufacturer.

### Analysis

The final classification of the patients resulted in 2 categories of IRIS and non-IRIS. Patients who interrupted ART >1 week, or stopped ART before 12 weeks, were excluded from the immunological analysis. Longitudinal percent levels of Tregs were analyzed using generalized estimating equations (equal correlation structure) adjusting for age, sex, ethnicity and viral loads. Cross-sectional analysis of baseline Treg levels was compared between the IRIS- and non-IRIS groups adjusted for the same possible confounders as mentioned above using the non-parametric Kruskal-Wallis test. Tests with a p-value <0.05 were considered statistically significant.

All analyses were performed in Stata (version 11) and figures were drawn using Prism (version 5).
